# Characteristics associated with comorbid lumbar spinal stenosis symptoms in people with knee or hip osteoarthritis: an analysis of 9,136 good life with osteoArthritis in Denmark (GLA:D®) participants

**DOI:** 10.1186/s12891-023-06356-3

**Published:** 2023-04-01

**Authors:** James J. Young, Alice Kongsted, Rikke Krüger Jensen, Ewa M. Roos, Carlo Ammendolia, Søren T. Skou, Dorte T. Grønne, Jan Hartvigsen

**Affiliations:** 1 Centre for Muscle and Joint Health, Department of Sports Science and Clinical Biomechanics, University of Southern Denmark, 55 Campusvej, Odense, 5230 Denmark; 2 Schroeder Arthritis Institute, Krembil Research Institute, University Health Network, Toronto, Canada; 3 Department of Research, Canadian Memorial Chiropractic College, Toronto, Canada; 4 Chiropractic Knowledge Hub, Odense, 5230 Denmark; 5 Rebecca MacDonald Centre for Arthritis and Autoimmune Diseases, Mount Sinai Hospital, Toronto, Canada; 6 Institute for Health Policy, Management and Evaluation, University of Toronto, Toronto, Canada; 7 The Research Unit PROgrez, Department of Physiotherapy and Occupational Therapy, Naestved-Slagelse-Ringsted Hospitals, Region Zealand, Slagelse, 4200 Denmark

**Keywords:** Lumbar spinal stenosis, Knee osteoarthritis, Hip osteoarthritis, Comorbidity, Association

## Abstract

**Background:**

Previous studies have found that lumbar spinal stenosis (LSS) often co-occurs with knee or hip OA and can impact treatment response. However, it is unclear what participant characteristics may be helpful in identifying individuals with these co-occurring conditions. The aim of this cross-sectional study was to explore characteristics associated with comorbid symptoms of lumbar spinal stenosis (LSS) in people with knee or hip osteoarthritis (OA) enrolled in a primary care education and exercise program.

**Methods:**

Sociodemographic, clinical characteristics, health status measures, and a self-report questionnaire on the presence of LSS symptoms was collected at baseline from the Good Life with osteoArthritis in Denmark primary care program for knee and hip OA. Cross-sectional associations between characteristics and the presence of comorbid LSS symptoms were assessed separately in participants with primary complaint of knee and hip OA, using domain-specific logistic models and a logistic model including all characteristics.

**Results:**

A total of 6,541 participants with a primary complaint of knee OA and 2,595 participants with a primary complaint of hip OA were included, of which 40% and 50% reported comorbid LSS symptoms, respectively. LSS symptoms were associated with similar characteristics in knee and hip OA. Sick leave was the only sociodemographic variable consistently associated with LSS symptoms. For clinical characteristics, back pain, longer symptom duration and bilateral or comorbid knee or hip symptoms were also consistently associated. Health status measures were not consistently related to LSS symptoms.

**Conclusion:**

Comorbid LSS symptoms in people with knee or hip OA undergoing a primary care treatment program of group-based education and exercise were common and associated with a similar set of characteristics. These characteristics may help to identify people with co-occurring LSS and knee or hip OA, which can be used to help guide clinical decision-making.

**Supplementary Information:**

The online version contains supplementary material available at 10.1186/s12891-023-06356-3.

## Background

Symptomatic lumbar spinal stenosis (LSS) is a common condition, affecting an estimated 11% of the general population and 25% of people in primary care settings [1]. LSS is most commonly a result of degenerative changes in the spine associated with ageing that cause narrowing of the spinal canal, resulting in spinal nerve ischemia and compression [2]. Symptoms of LSS include pain and neurological symptoms in the lower extremity [3] and functional limitations such as decreased walking capacity [4, 5].

Degenerative LSS is a consequence of lumbar spinal OA [6, 7], with the primary difference in clinical presentation compared to knee and hip OA being the potential neurological symptoms in the lower extremity. A growing body of evidence suggests that multi-joint OA, including lumbar spine OA or LSS, is more common than single-joint presentations [6, 8, 9]. A recent meta-analysis of co-occurring prevalence estimates found that symptomatic LSS occurs in up to 25% of people of with symptomatic knee OA and in up to 35% of people with symptomatic hip OA [10]. However, no included study investigated if people with co-occurring LSS and knee or hip OA have a different clinical presentation than those with these conditions in isolation, despite considerable overlap in the clinical presentations of LSS and knee and hip OA [11–14]. Since previous studies have shown comorbid LSS and other degenerative disorders of the lumbar spine affect OA treatment outcomes [15–21], a better understanding of the clinical presentation for co-occurring LSS with knee or hip OA can help inform clinical reasoning and shared-decision making when selecting management strategies, including potential prognostic significance.

A variety of symptoms associated with LSS are frequently reported by people with knee or hip OA participating in the Good Life with osteoArthritis in Denmark (GLA:D®) treatment program; a primary care treatment program consisting of group-based patient education and exercise therapy delivered across Denmark [22]. Participant data from the GLA:D® registry offers an opportunity to investigate factors associated with comorbid LSS symptoms as a necessary first step in determining potential risk factors for co-occurring presentations and improved identification of these individuals. The objective of this study was to explore sociodemographics, clinical characteristics, and health status measures that may be associated with comorbid symptoms of LSS in people with knee or hip OA enrolled in the GLA:D® primary care treatment program.

## Methods

### Study design

Cross-sectional analysis of baseline data from people with symptoms and functional limitations of knee or hip OA participating in a structured program of group education and supervised exercise (GLA:D®) [23]. Consecutive participants in the program between January 2019 to February 2020 with available baseline data on LSS symptoms were included. This study is reported according to the STROBE statement for observational studies.

### Participants

GLA:D® is a nationwide program in Denmark delivering group-based individualized education and exercise for people with knee and hip OA. Data from GLA:D® is collected via a combination of therapist-report and participant self-report [23]. People seeking care for knee and hip OA are eligible for GLA:D® if they can read and understand Danish and do not have a condition other than OA responsible for their joint symptoms (e.g. inflammatory joint disease, patellar tendinopathy) or with more severe symptoms not related to their OA (e.g. serious pathology, fibromyalgia) [23]. Detailed information about GLA:D® is available elsewhere [23].

Two cohorts of participants were identified at baseline according to their primary complaint: those with a primary knee complaint (knee cohort) and those with a primary hip complaint (hip cohort).

### Comorbid LSS symptoms

Participants in each cohort were considered to have comorbid LSS symptoms if they answered yes to the question “Do you sometimes feel pain or numbness in one/both legs or buttocks? (meaning other symptoms than from the knee or hip joint)” plus yes to at least one of the following in the last month: worsening when walking; worsening when standing; relieved when bending forwards; relieved when sitting; relieved when riding a bicycle; relieved when bending over a shopping cart; bending forward while walking; or feeling weakness in the legs while walking. All LSS symptom questions and outcome definitions are presented in Supplementary File 1. As there are no consensus diagnostic criteria for LSS [13, 24–26], these items were selected based on their common usage in diagnostic questionnaires and utility in identifying LSS-related leg pain from other sources of leg pain such as radiculopathy from lumbar disc herniation [27, 28]. Similar LSS definitions have been used in recent LSS clinical and epidemiological studies [21, 29, 30].

### Baseline characteristics

#### Sociodemographics

Sociodemographic items included age strata (< 50, 50–59, 60–69, 70–79, ≥ 80), sex (male/female), body mass index (BMI) categories (underweight [< 18.5 kg/m^2^], normal weight [18.5–24.9 kg/m^2^], overweight [25.0-29.9 kg/m^2^], obese [≥ 30.0 kg/m^2^]), highest level of education completed (primary school, secondary school, short-term education [< 3 years after secondary school], middle-term education [3–4 years after secondary school], long-term education [≥ 5 years after secondary school]), current employment (employed/student, sick leave full-time, sick leave part-time, retired, unemployed, self-imposed early retirement, early retirement due to low workability), and sick leave in past year due to knee/hip problems (yes/no).

#### Clinical characteristics

Symptom duration categories (< 3 months, 3–12 months, 13–24 months, > 24 months), presence of bilateral joint symptoms (yes/no), presence of hip symptoms (yes/no; knee cohort only) or presence of knee symptoms (yes/no; hip cohort only), presence of back pain in the past month (yes/no, dichotomized from pain numeric rating scale [0–10] using a cut-point of ≥ 1), and number of comorbidities categories (none, one, two, three or more) out of 13 conditions/disease categories collected (high blood pressure, high cholesterol, rheumatological diseases, osteoporosis, diabetes type 1, diabetes type 2, chronic heart failure, ischemic heart disease, anemia, stroke, Parkinson’s disease, dementia, other neurological diseases). Current use of pain medication (yes/no), current use of opioids (yes/no) and fear of movement (are you afraid that your joints will be damaged from physical activity and exercise (yes/no) were also included via self-report.

#### Health status measures

Patient-reported outcome measures included: Knee injury and Osteoarthritis Outcome Score 12-item version (KOOS-12) [31] or Hip disability and Osteoarthritis Outcome Score 12-item version (HOOS-12) [32] pain subscale (0 worst to 100 best), K/HOOS-12 function subscale (0 worst to 100 best), K/HOOS-12 quality of life subscale (0 worst to 100 best), Arthritis Self-Efficacy Scale (ASES) pain subscale (10 worst to 100 best) [33], ASES other symptoms subscale (10 worst to 100 best) [33] and University of California Los Angeles (UCLA) Activity Score (1 inactive to 10 active) [34]. Two objective measures of physical function were also included: 30-second chair-stand test (number of repetitions completed) and 40-meter fast-paced walk test (seconds) [35].

### Statistical analysis

Descriptive data for each cohort are presented as means or proportions and 95% confidence intervals (95% CI). Excluded participants with missing LSS symptom data were descriptively compared (means or proportions and 95% CI) on all available baseline data collected by the GLA:D® clinicians at time of enrollment: age strata, sex, BMI categories, symptom duration categories, pain medication use, and opioid use.

Baseline characteristics between participants reporting and not reporting LSS symptoms within each cohort were descriptively compared using means or proportions and 95% CI. No imputations of missing values were performed due to the low proportion of missing values and even distribution between those with and without LSS symptoms. To evaluate the association of baseline characteristics with LSS symptoms, four logistic regression models (LSS symptoms yes/no as the dependent variable) were built in the knee and hip cohorts, respectively. First, baseline characteristics for participants were entered into three domain-specific multivariable logistic models (sociodemographics model, clinical characteristics model, health status measures model). Then, all baseline characteristics were entered into one full multivariable logistic model.

The strength and significance of associations can change depending on the variables included in association models [36, 37]. Our domain-specific and full multivariable modelling strategy presents estimates from various combinations of independent variables and their subsequent effect on association estimates. Associations are reported as odds ratios (OR) and 95% CI. In all models, only variables with a variance inflation factor of less than four were included to avoid multicollinearity. All statistical analyses were performed in Stata 17.0 (StataCorp LLC, College Station, USA).

### Sensitivity analysis

In a sensitivity analysis we used an alternate outcome definition (Supplementary File 1) where comorbid LSS symptoms were defined as responding yes to “Do you sometimes feel pain or numbness in one/both legs or buttocks? (meaning other symptoms than from the knee or hip joint)”, plus yes to at least one worsening activity (walking or standing for a while) and at least one relieving activity (bending forwards; sitting; riding a bicycle; or bending over a shopping cart). This definition represents a more specific approach to defining comorbid LSS symptoms compared to the more sensitive primary outcome definition. The multivariable logistic regression analysis including all baseline characteristics (full model) was repeated for each cohort using the alternate LSS symptom definition as the independent variable.

### Sample size

Based on the previously published proportions of individuals self-reporting LSS symptoms in these cohorts [22] and an event-per-variable ratio of 15:1 [36], a minimum sample size of 834 knee participants and 655 hip participants were required for the largest multivariable model (full model; 22 total independent variables). Domain-specific models required a smaller minimum sample size.

## Results

### Sample characteristics

**Fig. 1:**
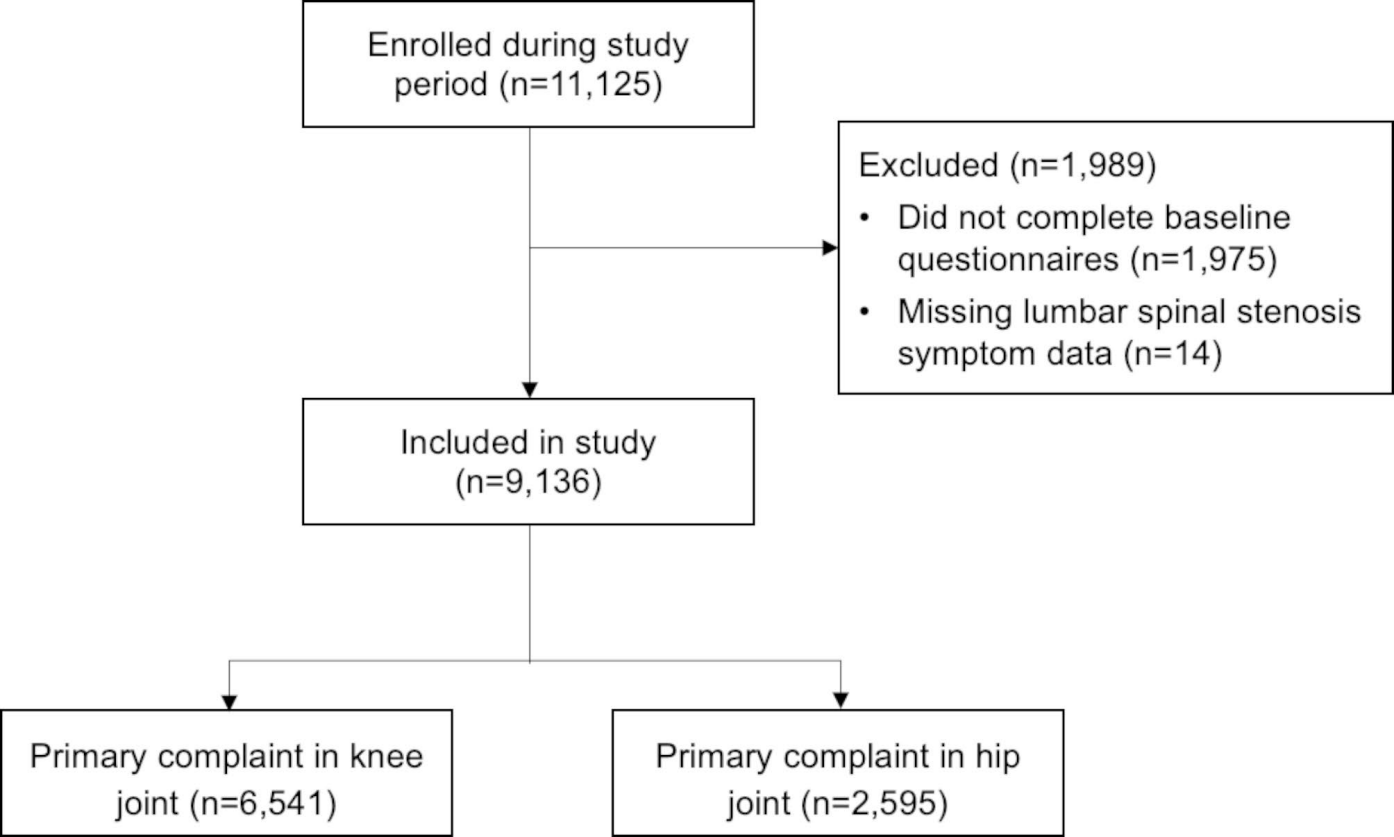
Study flow diagram

A total of 11,125 participants were enrolled in GLA:D® during the study period (Fig. 1). There were 6,541 participants with a primary complaint of knee OA and 2,595 with a primary complaint of hip OA who provided baseline data and answered the LSS symptom items and were included in the analysis (82% response rate) (Table 1). Participants who did not answer the LSS symptom items (n = 1,989) were more likely to be older, male, use pain medication, and use opioids (data not shown). There were only few missing data on baseline measures among included participants, except for on the 30-second chair-stand test (5.2% knee cohort; 4.7% hip cohort) and 40-meter fast-paced walk test (6.4% knee cohort; 6.4% hip cohort). Missing data on these measures was not associated with LSS symptom status in either cohort (data not shown).

**Table 1:** Sample baseline characteristics of participants with knee and hip osteoarthritis

	Knee cohort (n = 6,541)	Hip cohort (n = 2,595)
**Sociodemographics**		
Age, % (95% CI) < 50 50–59 60–69 70–79 ≥ 80 Missing, n	5.4 (4.9-6.0) 20.8 (19.8–21.8) 36.7 (35.6–37.9) 31.0 (29.9–32.1) 6.1 (5.5 6.7) 0	4.3 (3.6–5.2) 17.2 (15.8–18.7) 35.7 (33.9–37.6) 36.5 (34.7–38.4) 6.2 (5.3–7.2) 0
Female, % (95% CI) Missing, n	68.9 (67.8–70.0) 0	68.9 (67.0-70.7) 0
Body mass index, % (95% CI) Underweight Healthy weight Overweight Obese Missing, n	0.5 (0.3–0.7) 24.2 (23.1–25.2) 38.9 (37.7–40.1) 36.5 (35.3–37.7) 54	0.7 (4.7–1.2) 35.6 (33.7–37.4) 38.7 (36.9–40.6) 25.0 (23.3–26.7) 22
Education level, % (95% CI) Primary school Secondary school Short-term education Middle-term education Long-term education Missing, n	18.2 (17.3–19.2) 11.0 (10.3–11.8) 20.2 (19.2–21.2) 39.2 (38.0-40.4) 11.4 (10.7–12.2) 9	17.8 (16.4–19.3) 11.7 (10.5–13.0) 20.4 (18.9–22.0) 38.7 (36.8–40.6) 11.4 (10.3–12.7) 5
Current employment, % (95% CI) Employed/student Sick leave full-time Sick leave part-time Retired Unemployed Self-imposed early retirement Early retirement due to low workability Missing, n	32.3 (31.1–33.4) 2.6 (2.2-3.0) 2.7 (2.3–3.1) 53.6 (52.4–54.8) 2.1 (1.8–2.5) 4.4 (3.9–4.9) 2.4 (2.1–2.8) 0	29.7 (27.9–31.5) 1.5 (1.1–2.1) 2.2 (1.7–2.9) 59.3 (57.4–61.2) 1.3 (1.0-1.9) 3.4 (2.8–4.2) 2.4 (1.9–3.1) 0
Sick leave in past year, % (95% CI) Missing, n	11.5 (10.8–12.3) 1	6.6 (5.7–7.7) 0
**Clinical characteristics**		
Symptom duration, % (95%CI) < 3 months 3–12 months 13–24 months > 24 months Missing, n	8.7 (8.1–9.4) 46.0 (44.8–47.2) 15.4 (14.5–16.3) 29.9 (28.8–31.0) 2	5.4 (4.6, 6.4) 47.6 (45.7–49.5) 19.7 (18.2–21.3) 27.2 (25.5–29.0) 0
Bilateral joint symptoms, % (95% CI) Missing, n	43.7 (42.5–45.0) 1	24.6 (23.0-26.3) 0
Comorbid hip/knee symptoms, % (95% CI) Missing, n	18.6 (17.7–19.6) 3	35.1 (33.3–37.0) 0
Back pain in last month, % (95% CI) Missing, n	66.0 (64.8–67.1) 0	75.0 (73.3–76.7) 0
Number of comorbidities, % (95% CI) None One Two Three or more Missing, n	35.9 (34.7–37.1) 36.4 (35.3–37.6) 17.9 (17.0-18.9) 9.7 (9.0-10.5) 3	34.9 (33.1–36.8) 36.1 (34.2–38.0) 19.2 (17.7–20.8) 9.7 (8.6–10.9) 2
Pain medication use, % (95% CI) Missing, n	59.3 (58.1–60.4) 0	65.5 (63.7–67.4) 0
Opioid use, % (95% CI) Missing, n	4.6 (4.1–5.1) 1	6.7 (5.8–7.7) 0
Fear of movement, % (95% CI) Missing, n	15.3 (14.5–16.2) 0	10.3 (9.1–11.5) 0
**Health status measures**		
K/HOOS-12 pain subscale, mean (95% CI) Missing, n	50.3 (49.9–50.7) 0	49.3 (48.7–49.9) 0
K/HOOS-12 function subscale, mean (95% CI) Missing, n	56.9 (56.4–57.3) 0	59.4 (58.6–60.1) 0
K/HOOS-12 quality of life subscale, mean (95% CI) Missing, n	46.0 (45.6–46.4) 0	48.8 (48.1–49.4) 0
ASES pain subscale, mean (95% CI) Missing, n	64.8 (64.3–65.3) 10	61.2 (60.4–62.0) 5
ASES other symptoms subscale, mean (95% CI) Missing, n	69.1 (68.7–69.6) 10	67.0 (66.3–67.7) 5
UCLA Activity Score, mean (95% CI) Missing, n	5.5 (5.4–5.5) 0	5.6 (5.5–5.7) 0
30-second chair-stand test, mean (95% CI) Missing, n	11.9 (11.8–12.0) 339	12.2 (12.0-12.3) 123
40-meter fast-paced walk test, mean (95%) Missing, n	29.0 (28.7–29.2) 421	28.8 (28.4–29.1) 167

K/HOOS-12 (all subscales) scored 0(worst) to 100(best); ASES (all subscales) scored 10(worst) to 100(best); UCLA Activity Score scored 1(inactive) to 10(active); 30-second chair-stand test scored as number of repetitions completed; 40-meter fast-paced walk test scored in seconds

### Knee cohort

A total of 2,435 participants with a primary complaint of knee OA (37.2%) were classified as having comorbid LSS symptoms. Across the domain-specific and full models, obesity, sick leave due to knee/hip problems in the past year, back pain in the past month, three or more medical comorbidities, bilateral knee symptoms, comorbid hip symptoms, symptom duration > 24 months, and pain medication use were statistically significantly associated with reporting comorbid LSS symptoms (Table 2). People reporting LSS symptoms had slightly worse functional ability (KOOS-12 function subscale) and self-efficacy in managing other symptoms related to arthritis (ASES other symptoms subscale) compared to people not reporting LSS symptoms. Baseline characteristics in participants with and without comorbid LSS symptoms are presented in Supplementary File 1.

**Table 2:** Association of baseline characteristics with self-reported symptoms of lumbar spinal stenosis in participants with knee osteoarthritis

	Sociodemographics model, OR (95% CI)	Clinical characteristics model, OR (95% CI)	Health status measures model, OR (95% CI)	Full model, OR (95% CI)
**Sociodemographics**				
Age < 50 50–59 60–69 70–79 ≥ 80	Reference 0.98 (0.77–1.24) 0.79 (0.62 1.02) 0.75 (0.56-1.00)* 0.85 (0.60–1.20)			Reference 1.03 (0.79–1.34) 0.85 (0.64–1.12) 0.83 (0.60–1.14) 0.97 (0.66–1.42)
Female	1.09 (0.97–1.22)			0.99 (0.87–1.12)
Body mass index Underweight Healthy weight Overweight Obese	1.77 (0.85–3.70) Reference 1.34 (1.17–1.54)*** 1.52 (1.32–1.75)***			2.25 (1.00-5.06)* Reference 1.24 (1.07–1.44)** 1.18 (1.00-1.39)*
Education level Primary school Secondary school Short-term education Middle-term education Long-term education	Reference 0.89 (0.73–1.08) 0.86 (0.73–1.01) 0.91 (0.78–1.05) 0.73 (0.60–0.89)**			Reference 0.94 (0.76–1.16) 0.92 (0.77–1.11) 0.98 (0.84–1.56) 0.82 (0.66–1.02)
Current employment Employed/student Sick leave full-time Sick leave part-time Retired Unemployed Self-imposed early retirement Early retirement due to low workability	Reference 1.50 (1.08–2.07)* 1.38 (1.00-1.89)* 1.17 (0.98–1.39) 1.73 (1.22–2.46)** 1.18 (0.89–1.57) 2.33 (1.66–3.27)***			Reference 0.98 (0.68–1.40) 0.95 (0.67–1.34) 1.10 (0.91–1.34) 1.48 (1.01–2.17)* 1.20 (0.88–1.62) 1.55 (1.07–2.25)*
Sick leave in past year	1.41 (1.19–1.67)***			1.33 (1.10–1.60)**
**Clinical characteristics**				
Symptom duration < 3 months 3–12 months 13–24 months > 24 months		Reference 1.23 (1.00-1.50)* 1.30 (1.04–1.63)* 1.35 (1.09–1.66)**		Reference 1.23 (0.99–1.52) 1.23 (0.96–1.57) 1.32 (1.05–1.65)*
Bilateral knee symptoms		1.43 (1.29–1.60)***		1.40 (1.25–1.57)***
Comorbid hip symptoms		1.54 (1.34–1.76)***		1.54 (1.33–1.78)***
Back pain in last month		2.07 (1.84–2.33)***		1.86 (1.64–2.11)***
Number of comorbidities None One Two Three or more		Reference 1.12 (0.99–1.27) 1.35 (1.16–1.57)*** 1.82 (1.51–2.19)***		Reference 1.06 (0.92–1.21) 1.30 (1.10–1.53)** 1.60 (1.30–1.97)***
Pain medication use		1.41 (1.27–1.58)***		1.13 (1.00-1.28)*
Opioid use		1.41 (1.10–1.80)**		1.23 (0.94–1.60)
Fear of movement		1.36 (1.18–1.57)***		1.15 (0.98–1.35)
**Health status measures**				
KOOS-12 pain subscale			0.99 (0.99-1.00)*	1.00 (0.99-1.00)
KOOS-12 function subscale			0.99 (0.98–0.99)***	0.99 (0.98–0.99)***
KOOS-12 quality of life subscale			0.99 (0.99-1.00)**	1.00 (0.99-1.00)
ASES pain subscale			1.00 (1.00–1.00)	1.00 (1.00–1.00)
ASES other symptoms subscale			0.99 (0.98–0.99)***	0.99 (0.99-1.00)***
UCLA Activity Score			1.03 (1.00-1.06)	1.05 (1.01–1.08)**
30-second chair-stand test			1.01 (0.99–1.03)	1.02 (1.01–1.04)**
40-meter fast-paced walk test			1.00 (1.00-1.01)	1.00 (1.00-1.01)

* indicates p < 0.05; ** indicates p < 0.01; *** indicates p < 0.001; KOOS-12 (all subscales) scored 0(worst) to 100(best); ASES (all subscales) scored 10(worst) to 100(best); UCLA Activity Score scored 1(inactive) to 10(active); 30-second chair-stand test scored as number of repetitions completed; 40-meter fast-paced walk test scored in seconds

#### Sociodemographics

Four sociodemographic items were statistically significantly associated with LSS symptoms in both the domain-specific and full models: overweight (full model OR 1.24 [1.07–1.44]); obesity (full model OR 1.18 [1.00-1.39]); being unemployed (full model OR 1.48 [1.01–2.17]); and sick leave in the past year (full model OR 1.33 [1.10–1.60]). Obesity and sick leave in the past year remained significantly associated in the sensitivity analysis (Table 3).

**Table 3:** Association of baseline characteristics with self-reported symptoms of lumbar spinal stenosis in participants with hip osteoarthritis

	Sociodemographics model, OR (95% CI)	Clinical characteristics model, OR (95% CI)	Health status measures model, OR (95% CI)	Full model, OR (95% CI)
**Sociodemographics**				
Age < 50 50–59 60–69 70–79 ≥ 80	Reference 1.04 (0.68–1.59) 0.77 (0.50–1.18) 0.64 (0.40–1.03) 0.56 (0.32–0.98)*			Reference 1.04 (0.65–1.64) 0.75 (0.47–1.20) 0.63 (0.37–1.06) 0.47 (0.25–0.88)*
Female	0.96 (0.81–1.15)			0.93 (0.77–1.13)
Body mass index Underweight Healthy weight Overweight Obese	0.58 (0.22–1.55) Reference 1.06 (0.88–1.28) 1.18 (0.96–1.46)			0.61 (0.22–1.72) Reference 0.98 (0.80–1.20) 0.93 (0.73–1.19)
Education level Primary school Secondary school Short-term education Middle-term education Long-term education	Reference 0.87 (0.64–1.17) 0.97 (0.75–1.25) 0.90 (0.72–1.13) 0.81 (0.59–1.09)			Reference 0.96 (0.69–1.33) 1.10 (0.83–1.46) 1.12 (0.87–1.44) 0.99 (0.70–1.39)
Current employment Employed/student Sick leave full-time Sick leave part-time Retired Unemployed Self-imposed early retirement Early retirement due to low workability	Reference 0.79 (0.41–1.52) 1.73 (0.95–3.15) 1.06 (0.81–1.39) 1.77 (0.84–3.75) 1.06 (0.66–1.69) 1.01 (0.60–1.71)			Reference 0.78 (0.37–1.62) 1.15 (0.60–2.21) 0.95 (0.71–1.27) 1.20 (0.53–2.69) 1.01 (0.60–1.68) 0.60 (0.34–1.07)
Sick leave in past year	1.90 (1.34–2.72)***			1.48 (1.01–2.17)*
**Clinical characteristics**				
Symptom duration < 3 months 3–12 months 13–24 months > 24 months		Reference 1.80 (1.23–2.63)** 1.99 (1.33–2.98)** 2.05 (1.38–3.03)***		Reference 1.85 (1.22–2.79)** 2.01 (1.30–3.12)** 2.00 (1.30–3.08)**
Bilateral hip symptoms		1.41 (1.17–1.70)***		1.27 (1.04–1.55)*
Comorbid knee symptoms		1.22 (1.03–1.44)*		1.14 (0.95–1.37)
Back pain in last month		1.74 (1.44–2.10)***		1.42 (1.15–1.74)**
Number of comorbidities None One Two Three or more		Reference 1.03 (0.85–1.24) 1.18 (0.94–1.48) 1.57 (1.17–2.11)**		Reference 1.09 (0.88–1.33) 1.25 (0.97–1.62) 1.62 (1.16–2.28)**
Pain medication use		1.41 (1.19–1.68)***		1.26 (1.04–1.53)*
Opioid use		1.48 (1.06–2.06)*		1.24 (0.86–1.77)
Fear of movement		1.58 (1.21–2.05)**		1.30 (0.97–1.73)
**Health status measures**				
HOOS-12 pain subscale			0.99 (0.99-1.00)	1.00 (0.99–1.01)
HOOS-12 function subscale			0.99 (0.98-1.00)**	0.99 (0.98-1.00)**
HOOS-12 quality of life subscale			0.99 (0.98-1.00)*	0.99 (0.99-1.00)
ASES pain subscale			0.99 (0.99-1.00)	1.00 (0.99-1.00)
ASES other symptoms subscale			0.99 (0.99-1.00)*	0.99 (0.99-1.00)
UCLA Activity Score			1.05 (1.00-1.10)	1.04 (0.99–1.10)
30-second chair-stand test			0.99 (0.97–1.02)	1.00 (0.98–1.03)
40-meter fast-paced walk test			0.99 (0.98-1.00)	1.00 (0.99–1.02)

* indicates p < 0.05; ** indicates p < 0.01; *** indicates p < 0.001; HOOS-12 (all subscales) scored 0(worst) to 100(best); ASES (all subscales) scored 10(worst) to 100(best); UCLA Activity Score scored 1(inactive) to 10(active); 30-second chair-stand test scored as number of repetitions completed; 40-meter fast-paced walk test scored in seconds

#### Clinical characteristics

Seven clinical characteristics were associated with LSS symptoms in both the domain-specific and full models: back pain in last month (full model OR 1.86 [1.64–2.11]); two medical comorbidities (full model OR 1.30 [1.10–1.53]); three or more medical comorbidities (full model OR 1.60 [1.30–1.97]); bilateral knee symptoms (full model OR 1.40 {1.25–1.57]); comorbid hip symptoms (full model OR 1.54 [1.33–1.78]); symptom duration > 24 months (full model OR 1.32 [1.05–1.65]); and pain medication use (full model OR 1.13 [1.00-1.28]). Back pain in the last month, three or more medical comorbidities, bilateral knee symptoms, comorbid hip symptoms, symptom duration > 24 months, and pain medication use remained significantly associated with LSS symptoms in the sensitivity analysis (Table 3).

#### Health status measures

Better scores on two health status measures were associated with a slightly reduced likelihood of LSS symptoms: KOOS-12 function subscale (full model OR 0.99 [0.98–0.99]); and ASES other symptoms subscale (full model OR 0.99 [0.99-1.00]). Better scores on both measures remained significantly associated with a reduced likelihood of LSS in the sensitivity analysis (Table 3).

### Hip cohort

A total of 1,253 participants with a primary complaint of hip OA (48.2%) were classified as having comorbid LSS symptoms. Across the domain-specific and full models sick leave in the past year, back pain in the past month, a symptom duration of 3–12 months, 13–24 months, or > 24 months were associated with reporting comorbid LSS symptoms (Table 4). People reporting LSS symptoms had slightly worse functional ability (HOOS-12 function subscale). Baseline characteristics in participants with and without comorbid LSS symptoms are presented in Supplementary File 1.

**Table 4:** Association of baseline characteristics with alternate lumbar spinal stenosis symptom definition in participants with knee and hip osteoarthritis

	Knee cohort		Hip cohort	
	**Primary LSS symptom model**, OR (95% CI)	**Alternate LSS symptom model**, OR (95% CI)	**Primary LSS symptom model**, OR (95% CI)	**Alternate LSS symptom model**, OR (95% CI)
**Sociodemographics**				
Age < 50 50–59 60–69 70–79 ≥ 80	Reference 1.03 (0.79–1.34) 0.85 (0.64–1.12) 0.83 (0.60–1.14) 0.97 (0.66–1.42)	Reference 1.22 (0.91–1.64) 0.97 (0.71–1.32) 0.92 (0.64–1.32) 1.06 (0.79–1.64)	Reference 1.04 (0.65–1.64) 0.75 (0.47–1.20) 0.63 (0.37–1.06) 0.47 (0.25–0.88)*	Reference 1.17 (0.73–1.90) 1.02 (0.62–1.67) 0.74 (0.43–1.29) 0.62 (0.31–1.22)
Female	0.99 (0.87–1.12)	0.97 (0.84–1.12)	0.93 (0.77–1.13)	0.94 (0.76–1.15)
Body mass index Underweight Healthy weight Overweight Obese	2.25 (1.00-5.06)* Reference 1.24 (1.07–1.44)** 1.18 (1.00-1.39)*	2.18 (0.90–5.26) Reference 1.12 (0.94–1.34) 1.32 (1.10–1.58)**	0.61 (0.22–1.72) Reference 0.98 (0.80–1.20) 0.93 (0.73–1.19)	1.07 (0.36–3.18) Reference 1.08 (0.86–1.34) 1.09 (0.84–1.40)
Education level Primary school Secondary school Short-term education Middle-term education Long-term education	Reference 0.94 (0.76–1.16) 0.92 (0.77–1.11) 0.98 (0.84–1.56) 0.82 (0.66–1.02)	Reference 0.83 (0.65–1.05) 0.96 (0.79–1.18) 0.97 (0.81–1.15) 0.82 (0.64–1.06)	Reference 0.96 (0.69–1.33) 1.10 (0.83–1.46) 1.12 (0.87–1.44) 0.99 (0.70–1.39)	Reference 1.21 (0.86–1.70) 1.06 (0.78–1.43) 1.21 (0.93–1.59) 1.00 (0.68–1.45)
Current employment Employed/student Sick leave full-time Sick leave part-time Retired Unemployed Self-imposed early retirement Early retirement due to low workability	Reference 0.98 (0.68–1.40) 0.95 (0.67–1.34) 1.10 (0.91–1.34) 1.48 (1.01–2.17)* 1.20 (0.88–1.62) 1.55 (1.07–2.25)*	Reference 0.84 (0.57–1.24) 0.93 (0.64–1.34) 1.21 (0.97–1.51) 1.44 (0.97–2.14) 1.15 (0.81–1.63) 1.22 (0.83–1.79)	Reference 0.78 (0.37–1.62) 1.15 (0.60–2.21) 0.95 (0.71–1.27) 1.20 (0.53–2.69) 1.01 (0.60–1.68) 0.60 (0.34–1.07)	Reference 0.75 (0.36–1.57) 0.93 (0.51–1.70) 1.04 (0.76–1.42) 1.09 (0.49–2.42) 1.01 (0.58–1.74) 0.72 (0.39–1.30)
Sick leave in past year	1.33 (1.10–1.60)**	1.29 (1.06–1.58)*	1.48 (1.01–2.17)*	1.51 (1.04–2.18)*
**Clinical characteristics**				
Symptom duration Less than 3 months 3–12 months 13–24 months > 24 months	Reference 1.23 (0.99–1.52) 1.23 (0.96–1.57) 1.32 (1.05–1.65)*	Reference 1.60 (1.22–2.08)** 1.69 (1.26–2.27)*** 1.61 (1.22–2.12)**	Reference 1.85 (1.22–2.79)** 2.01 (1.30–3.12)** 2.00 (1.30–3.08)**	Reference 1.86 (1.16-3.00)* 1.92 (1.16–3.17)* 1.75 (1.07–2.87)*
Bilateral joint symptoms	1.40 (1.25–1.57)***	1.27 (1.11–1.45)***	1.27 (1.04–1.55)*	1.10 (0.89–1.36)
Comorbid hip or knee symptoms	1.54 (1.33–1.78)***	1.46 (1.24–1.70)***	1.14 (0.95–1.37)	1.14 (0.94–1.39)
Back pain in last month	1.86 (1.64–2.11)***	1.94 (1.67–2.26)***	1.42 (1.15–1.74)**	1.55 (1.23–1.96)***
Number of comorbidities None One Two Three or more	Reference 1.06 (0.92–1.21) 1.30 (1.10–1.53)** 1.60 (1.30–1.97)***	Reference 1.04 (0.89–1.22) 1.13 (0.94–1.37) 1.52 (1.22–1.89)***	Reference 1.09 (0.88–1.33) 1.25 (0.97–1.62) 1.62 (1.16–2.28)**	Reference 1.00 (0.79–1.25) 1.26 (0.97–1.65) 1.38 (0.97–1.94)
Pain medication use	1.13 (1.00-1.28)*	1.27 (1.10–1.46)**	1.26 (1.04–1.53)*	1.21 (0.98–1.49)
Opioid use	1.23 (0.94–1.60)	1.22 (0.93–1.59)	1.24 (0.86–1.77)	0.98 (0.68–1.40)
Fear of movement	1.15 (0.98–1.35)	1.16 (0.98–1.38)	1.30 (0.97–1.73)	1.01 (0.75–1.37)
**Health status measures**				
K/HOOS-12 pain subscale	1.00 (0.99-1.00)	1.00 (0.99-1.00)	1.00 (0.99–1.01)	1.00 (0.99–1.01)
K/HOOS-12 function subscale	0.99 (0.98–0.99)***	0.99 (0.98–0.99)***	0.99 (0.98-1.00)**	0.99 (0.98–0.99)***
K/HOOS-12 quality of life subscale	1.00 (0.99-1.00)	0.99 (0.99-1.00)*	0.99 (0.99-1.00)	0.99 (0.98-1.00)*
ASES pain subscale	1.00 (1.00–1.00)	1.00 (1.00-1.01)	1.00 (0.99-1.00)	1.00 (0.99–1.01)
ASES other symptoms subscale	0.99 (0.99-1.00)***	0.99 (0.99-1.00)*	0.99 (0.99-1.00)	0.99 (0.98-1.00)*
UCLA Activity Score	1.05 (1.01–1.08)**	1.07 (1.03–1.11)***	1.04 (0.99–1.10)	1.01 (0.96–1.07)
30-second chair-stand test	1.02 (1.01–1.04)**	1.01 (0.99–1.03)	1.00 (0.98–1.03)	1.01 (0.98–1.04)
40-meter fast-paced walk test	1.00 (1.00-1.01)	1.01 (1.00-1.01)	1.00 (0.99–1.02)	1.01 (0.99–1.02)

Odds ratios from primary analysis (full models) are again presented here to aid in interpretation; * indicates p < 0.05; ** indicates p < 0.01; *** indicates p < 0.001; K/HOOS-12 (all subscales) scored 0(worst) to 100(best); ASES (all subscales) scored 10(worst) to 100(best); UCLA Activity Score scored 1(inactive) to 10(active); 30-second chair-stand test scored as number of repetitions completed; 40-meter fast-paced walk test scored in seconds

#### Sociodemographics

The only sociodemographic item associated with LSS symptoms in both the domain-specific and full models was sick leave in the past year (full model OR 1.48 [1.01–2.17]). Additionally, being ≥ 80 years old was associated with a reduced likelihood of LSS symptoms (full model OR 0.47 [0.25–0.88]). Sick leave in the past year remained significantly associated with LSS symptoms in the sensitivity analysis (Table 3).

#### Clinical characteristics

Seven clinical characteristics were associated with LSS symptoms in both the domain-specific and full models: back pain in last month (full model OR 1.42 [1.15–1.74]); symptom duration 3–12 months (full model OR 1.85 [1.22–2.79]); symptom duration 13–24 months (full model OR 2.01 [1.30–3.12]); symptom duration > 24 months (full model OR 2.00 [1.30–3.08]); bilateral hip symptoms (full model OR 1.27 [1.04–1.55]); three or more medical comorbidities (full model OR 1.62 [1.16–2.28]); and pain medication use (full model OR 1.62 [1.16–2.28]). Only back pain in the last month and symptom durations of 3–12 months, 13–24 months, and > 24 months remained significantly associated with LSS symptoms in the sensitivity analysis (Table 3).

#### Health status measures

A better score on the HOOS-12 function subscale was associated with a slightly reduced likelihood of LSS symptoms in both models (full model OR 0.99 [0.98-1.00]). Better scores on the HOOS-12 function subscale remained significantly associated with a reduced likelihood of LSS symptoms in the sensitivity analysis (Table 3).

## Discussion

This was the first study to investigate characteristics related to reporting comorbid LSS symptoms in people with knee or hip OA. Approximately one-third of people with knee OA and one-half of people with hip OA also reported LSS symptoms and a similar pattern of associated characteristics was found for both cohorts. Sick leave in the past year was the only sociodemographic item consistently associated with LSS symptoms. Clinical characteristics such as back pain, longer durations of knee or hip symptoms, multiple affected knee or hip joints, and higher numbers of medical comorbidities had the most consistent association with LSS symptoms, whereas health status measures were not consistently related to LSS symptoms. These characteristics may be helpful in identifying comorbid LSS symptoms in people with knee or hip OA in a primary care setting.

### Sociodemographics

Participants with co-occurring LSS symptoms and knee or hip OA are more often on sick leave, indicating a more severe disease profile. Interestingly in the knee cohort, work absence (unemployed and early retirement due to low workability) was also associated with increased odds of LSS symptoms, but not when using the alternate LSS symptom outcome. Additionally, being on sick leave (full- or part-time) was associated in the domain-specific model, but not in the full model. These findings, although inconsistent, are in line with previous literature that co-occurring musculoskeletal conditions are associated with poorer work-related outcomes [38, 39].

In the hip cohort only, reduced odds of LSS symptoms were found in people in the oldest age strata (≥ 80 years), which is surprising considering that the prevalence of LSS [1, 40] and multimorbidity [41] increases with age. A possible explanation is selection bias in GLA:D® where older adults enrolled in an exercise program are likely in better overall health compared to their population peers who do not enroll and therefore less likely to have comorbid LSS. Alternatively, this finding may indicate a limitation in the validity of the LSS symptom definition since this associations was not confirmed in the sensitivity analysis. It may also be that as individuals age, they are less likely to engage in activities included in the LSS symptom items, but this hypothesis requires further evaluation.

### Clinical characteristics

In both cohorts, back pain in the last month was associated (OR ranging from 1.42 to 2.07) with LSS symptoms independent of the definition used, which is likely a result of the relationship between LSS and back pain [42]. However, low back pain is not considered necessary in the diagnosis of LSS [25, 26] because some people with LSS do not experience low back pain. This may explain why not all individuals in our study with LSS symptoms report back pain. Low back pain is also a common comorbidity in people with knee or hip OA [43, 44], with a prevalence of 67% and 75% among knee and hip participants in GLA:D® [45]. Our study findings suggest it is likely that a proportion of people with knee or hip OA are experiencing back pain related to LSS.

A greater number of symptomatic knee and hip joints (i.e., bilateral knee/hip and/or comorbid hip/knee involvement) are likely related to co-occurring LSS in people with knee or hip OA, which may be part of a multi-joint OA presentation. We found that participants with bilateral symptoms in the hip cohort and participants with comorbid hip symptoms in the knee cohort were more likely to report LSS symptoms than those reporting unilateral hip symptoms (hip cohort) and no comorbid hip symptoms (knee cohort). We also found bilateral knee pain in the knee cohort was consistently associated with LSS symptoms and evidence of an association with comorbid knee symptoms in the hip cohort (domain-specific model only). The overall findings in both cohorts suggest that the more symptomatic joints (bilateral knee or hip and comorbid knee or hip), the more likely that there are also LSS symptoms.

It is unclear if comorbid LSS is part of a multi-joint OA presentation or a widespread pain presentation. A relationship between hip OA and LSS is proposed in the hip-spine syndrome literature [14, 19, 46] and previous studies have suggested spinal OA/LSS be considered in multi-joint OA definitions [6, 8]. However, our findings may also be explained by the symptomatic overlap in hip OA and LSS [11–13] or via widespread pain or central sensitization mechanism in people with OA [47]. Our findings of a consistent association between comorbid LSS symptoms and longer symptom durations in people with knee or hip OA (> 24 months) might support the hypothesis that widespread pain has developed and is partially responsible for the observed association between hip or knee OA and LSS symptoms.

It is likely that an increasing number of comorbidities is a risk factor for co-occurring knee or hip OA and LSS. People with higher levels of multimorbidity are at risk of developing further conditions and there is a known relationship between musculoskeletal disorders and other non-communicable diseases [48]. We found that having three or more medical comorbidities was consistently associated with LSS symptoms in the knee cohort and inconsistently associated in the hip cohort. There was also an inconsistent association with two medical comorbidities in the knee cohort.

### Health status measures

Most health status measures were not associated with comorbid LSS symptoms, except for higher levels of knee-specific (KOOS-12) or hip-specific (HOOS-12) functional ability and reduced odds of LSS symptoms. However, the odds of reporting LSS symptoms decreased by only 0.01 per unit increase in functional ability in both cohorts, which is unlikely to be a clinically relevant association. Moreover, the absence of meaningful associations between the objective physical function tests and LSS symptoms in both cohorts further support the unlikely relationship between functional ability and LSS symptoms in people with knee or hip pain. The similar functional impairments found in primary care patients with OA and LSS [2, 4] may explain why significantly worse scores on functional measures were not associated with LSS symptoms in our sample.

### Study limitations

The primary limitation of this study is the unknown validity of the LSS symptom definitions. Our definition may not be specific to LSS, potentially identifying participants with leg pain due to non-specific low back pain and/or lumbar disc herniation. However, there is no consensus regarding the best method to diagnose LSS [13, 24–26]. The symptom items used in this study are commonly associated with LSS [11, 25, 26] and have been shown to clinically differentiate LSS from other causes of back-related leg pain [27]. While two systematic reviews have shown that the symptom items used in our study are commonly used in the available published literature and can be helpful to identify LSS, they also found that proper studies to determine the accuracy of these items are lacking [28] and they may only have moderate associations with LSS [27]. It is also unknown if these self-report questions can differentiate symptoms of LSS from those in knee or hip OA, which is especially problematic when trying to differentiate LSS and hip OA [11, 13]. LSS symptoms can be variable [2] and thus, all participants with LSS symptoms may not have been captured by our definition. Additionally, participants were instructed to answer the LSS symptom items in regards to symptoms that were different from their knee or hip complaint, and we conducted a sensitivity analysis using an alternate LSS symptom definition. Finally, we expect the term “numbness” in the symptom items to be novel and appreciably different for people with knee or hip OA, and therefore more specific to LSS symptoms.

Selection bias may also play a role as we only included people seeking care for their knee or hip OA and due to the criteria requiring that GLA:D® participants do not have additional conditions that (1) are responsible for their joint condition or (2) have more severe symptoms [23]. We therefore expect that people with more severe presentations of LSS had been offered alternative interventions by the enrolling GLA:D® clinicians. Missing baseline data may also introduce selection bias in the cohorts. The included participants were younger, had a higher proportion of females, and were less likely to be using pain medication and opioids compared to those not answering the LSS symptom item. This may bias the analysis sample towards less severe comorbid LSS symptoms. Overall, a study sample biased towards less severe comorbid LSS symptoms, may result in increased uncertainty in associations with participant characteristics and hence we probably did not overestimate associations.

### Study strengths

This was the first study to investigate patient characteristics associated with comorbid LSS symptoms in people with knee or hip OA. We used a pragmatic and low resource approach to develop initial association estimates in a real-world clinical setting. The large sample size in both cohorts allowed us to investigate a wide range of patient characteristics. Additionally, the large sample size likely means that any uncertainty around association estimates reflects true uncertainty in identifying LSS and is not due to a lack of participants, increasing our confidence in the results. Overall, the findings of this study raise awareness of comorbid LSS in patients with knee or hip OA, which with further research may help clinicians identify these patients and aid in treatment decision-making.

### Future research and implications

All future investigations will benefit from the development of a standard and valid set of LSS symptoms, an agreed-upon case definition of symptomatic LSS, and methods to aid in the clinical differentiation between LSS and knee and hip OA. At minimum, more robust methods for defining the presence of LSS, such as a clustering of clinical symptoms and confirmatory imaging, should be developed. It would also be beneficial to explore how LSS-related characteristics, such as number of stenotic segments, symptom duration, and symptom severity measures are related to the presence of co-occurring LSS and knee and hip OA.

Our results should inform prospective studies or analyses of other available data sources to validate these findings. Identification of such risk factors for the development of LSS or lower extremity OA in these populations, or the identification of people developing LSS and knee or hip OA simultaneously, may help inform care planning.

It is possible that characteristics associated with comorbid presentations differ between populations with primary complaints of LSS or knee or hip OA. Investigations for these factors in populations with differing severity levels of LSS is also needed, since the prevalence of LSS increases drastically across community samples, primary care, and secondary care [1] and may impact treatment decisions differently depending on the care setting. Finally, further research on the impact of multimorbid presentations on treatment response is needed. There is evidence to suggest people with multimorbid LSS with knee or hip OA experience poorer surgical outcomes [6, 15, 18, 49–51], but little is known about the response to other recommended interventions for OA and LSS such as education and exercise.

## Conclusion

Comorbid LSS symptoms in people with knee or hip OA undergoing a primary care treatment program of group-based education and exercise were common and were associated with similar baseline characteristics. These characteristics may help to identify people with co-occurring LSS and knee or hip OA and help guide treatment decision-making.

## Electronic supplementary material

Below is the link to the electronic supplementary material.

Supplementary Material 1

## Data Availability

The datasets generated and/or analysed during the current study are not publicly available due to licensing restrictions on availability of data, but are available from the corresponding author upon reasonable request with applicable permissions.

## List of abbreviations

LSS: lumbar spinal stenosis
OA: osteoarthritis
GLA:D®: Good Life with osteoArthritis in Denmark
STROBE: Strengthening the Reporting of Observational studies in Epidemiology
KOOS-12: Knee injury and Osteoarthritis Outcome Score 12-item version
HOOS-12: Hip disability and Osteoarthritis Outcome Score 12-item version
ASES: Arthritis Self-Efficacy Scale
UCLA: University of California Los Angeles.
