# Supplementary material for: Characteristics associated with comorbid lumbar spinal stenosis symptoms in people with knee or hip osteoarthritis: an analysis of 9,136 good life with osteoArthritis in Denmark (GLA:D®) participants
Source: BMC Musculoskelet Disord. 2023 Apr 1;24:250. doi: 10.1186/s12891-023-06356-3 (PMC10067254; doi:10.1186/s12891-023-06356-3)
Supplement: Supplementary file 1 — Supplementary Material 1 [file 12891_2023_6356_MOESM1_ESM.docx]

**Supplementary File 1**

**LSS symptom items**

All questions on the LSS symptom questionnaire use self-reported answers by the participant with ”yes” or ”no” response options.

1. Do you sometimes have pain or numbness in one/both legs or buttocks? [other symptoms unrelated to knee or hip]
2. Do you feel pain or numbness in both legs or buttocks? (please mark yes if you had the symptom during the last month)
3. Do you have numbness under both feet? (please mark yes if you had the symptom during the last month)
4. Is your pain or numbness in one or both legs or buttocks worsening when you are walking? (please mark yes if you had the symptom during the last month)
5. Is your pain or numbness in one or both legs or in the buttock worsening when you have been standing for a while?(please mark yes if you had the symptom during the last month)
6. Is your pain or numbness in one or both legs or in the buttock relieved when you are bending forwards? (please mark yes if you had the symptom during the last month)
7. Is your pain or numbness in one or both legs or in the buttock relieved when you are sitting? (please mark yes if you had the symptom during the last month)
8. Is your pain or numbness in one or both legs or in the buttock relieved when you are riding a bicycle? (please mark yes if you had the symptom during the last month)
9. Is your pain or numbness in one or both legs or in the buttock relieved when you are bending over the shopping cart? (please mark yes if you had the symptom during the last month)
10. Do you bend forward while walking? (please mark yes if you had the symptom during the last month)
11. Do you have the feeling of weakness in your legs while walking? (please mark yes if you had the symptom during the last month)

**Primary analysis outcome definition**

In the primary analysis, participants were considered to have comorbid LSS symptoms if answering yes to:

1. Do you sometimes have pain or numbness in one/both legs or buttocks? [other symptoms unrelated to knee or hip],

plus yes to at least one of the following:

4. Is your pain or numbness in one or both legs or buttocks worsening when you are walking? (please mark yes if you had the symptom during the last month)

5. Is your pain or numbness in one or both legs or in the buttock worsening when you have been standing for a while? (please mark yes if you had the symptom during the last month)

6. Is your pain or numbness in one or both legs or in the buttock relieved when you are bending forwards? (please mark yes if you had the symptom during the last month)

7. Is your pain or numbness in one or both legs or in the buttock relieved when you are sitting? (please mark yes if you had the symptom during the last month)

8. Is your pain or numbness in one or both legs or in the buttock relieved when you are riding a bicycle? (please mark yes if you had the symptom during the last month)

9. Is your pain or numbness in one or both legs or in the buttock relieved when you are bending over the shopping cart? (please mark yes if you had the symptom during the last month)

10. Do you bend forward while walking? (please mark yes if you had the symptom during the last month)

11. Do you have the feeling of weakness in your legs while walking? (please mark yes if you had the symptom during the last month).

**Sensitivity analysis outcome definition** (alternate LSS symptom definition)

In the sensitivity analysis, participants were considered to have comorbid LSS symptoms if answering yes to:

1. Do you sometimes have pain or numbness in one/both legs or buttocks? [other symptoms unrelated to knee or hip],

plus yes to at least one of the following:

4. Is your pain or numbness in one or both legs or buttocks worsening when you are walking? (please mark yes if you had the symptom during the last month)

5. Is your pain or numbness in one or both legs or in the buttock worsening when you have been standing for a while? (please mark yes if you had the symptom during the last month),

plus yes to at least one of the following:

6. Is your pain or numbness in one or both legs or in the buttock relieved when you are bending forwards? (please mark yes if you had the symptom during the last month)

7. Is your pain or numbness in one or both legs or in the buttock relieved when you are sitting? (please mark yes if you had the symptom during the last month)

8. Is your pain or numbness in one or both legs or in the buttock relieved when you are riding a bicycle? (please mark yes if you had the symptom during the last month)

9. Is your pain or numbness in one or both legs or in the buttock relieved when you are bending over the shopping cart? (please mark yes if you had the symptom during the last month).

# **Supplementary tables**

**Table S1.** Baseline characteristics for knee osteoarthritis participants with and without comorbid lumbar spinal stenosis symptoms based on primary analysis outcome definition.

|  | **Symptoms of LSS** (n=2,435) | **No symptoms of LSS** (n=4,106) |
| --- | --- | --- |
| **Social demographics** |  |  |
| Age, % (95% CI)  <50  50-59  60-69  70-79  ≥80  Missing, n | 6.4 (5.5-7.5)  23.6 (21.9-25.3)  35.6 (33.7-37.6)  28.5 (26.7-30.3)  5.9 (5.0-6.9)  0 | 4.8 (4.2-5.5)  19.1 (17.9-20.3)  37.4 (35.9-38.9)  32.5 (31.1-33.9)  6.2 (5.5-7.0)  0 |
| Female, % (95% CI)  Missing, n | 70.3 (68.4-72.1)  0 | 68.1 (66.7-69.6)  0 |
| Body mass index, % (95% CI)  Underweight  Healthy weight  Overweight  Obese  Missing, n | 0.5 (0.3-0.9)  19.4 (17.9-21.1)  38.5 (36.6-40.5)  41.0 (39.1-43.0)  12 | 0.4 (0.2-0.7)  26.6 (25.3-28.0)  38.6 (37.1-40.1)  33.3 (31.9-34.8)  42 |
| Education level, % (95% CI)  Primary school  Secondary school  Short-term education  Middle-term education  Long-term education  Missing, n | 19.8 (18.3-21.5)  11.2 (9.9-12.5)  20.5 (18.9-22.1)  39.4 (37.4-41.4)  9.3 (8.2-10.5)  0 | 17.2 (16.1-18.4)  10.9 (10.0-11.9)  19.9 (18.7-21.2)  39.0 (37.5-40.5)  12.7 (11.7-13.7)  9 |
| Current employment, % (95% CI)  Employed/student  Sick leave full-time  Sick leave part-time  Retired  Unemployed  Self-imposed early retirement  Early retirement due to low workability  Missing, n | 31.8 (30.0-33.7)  3.5 (2.8-4.3)  3.5 (2.8-4.3)  50.2 (48.2-52.2)  3.0 (2.3-3.7)  4.3 (3.5-5.2)  3.7 (3.0-4.5)  0 | 32.5 (31.1-34.0)  2.0 (1.6-2.4)  2.2 (1.8-2.7)  55.7 (54.1-57.2)  1.6 (1.2-2.0)  4.4 (3.8-5.1)  1.7 (1.3-2.1)  0 |
| Sick leave in past year, % (95% CI)  Missing, n | 14.9 (13.5-16.3)  0 | 9.5 (8.6-10.5)  1 |
| **Clinical characteristics** |  |  |
| Symptom duration, % (95%CI)  Less than 3 months  3-12 months  13-24 months  More than 24 months  Missing, n | 7.0 (6.0-8.1)  44.1 (42.1-46.1)  16.0 (14.5-17.5)  32.9 (31.1-34.8)  0 | 9.7 (8.9-10.7)  47.2 (45.6-48.7)  15.0 (13.9-16.1)  28.1 (26.7-29.5)  2 |
| Bilateral knee symptoms, % (95% CI)  Missing, n | 49.6 (47.6-51.6)  0 | 40.3 (38.8-41.8)  1 |
| Comorbid hip symptoms, % (95% CI)  Missing, n | 23.7 (22.1-25.5)  0 | 15.6 (14.5-16.7)  3 |
| Back pain in last month, % (95% CI)  Missing, n | 77.9 (76.2-79.5)  0 | 58.9 (57.4-60.4)  0 |
| Number of comorbidities, % (95% CI)  None  One  Two  Three or more  Missing, n | 30.9 (29.1-32.8)  35.1 (33.2-37.0)  20.4 (18.8-22.1)  13.6 (12.3-15.0)  0 | 38.8 (37.3-40.3)  37.2 (35.8-38.7)  16.4 (15.3-17.6)  7.4 (6.6-8.3)  3 |
| Pain medication use, % (95% CI)  Missing, n | 66.6 (64.7-68.5)  0 | 54.9 (53.4-56.5)  0 |
| Opioid use, % (95% CI)  Missing, n | 6.7 (5.7-7.8)  0 | 3.3 (2.8-3.9)  1 |
| Fear of movement, % (95% CI)  Missing, n | 18.0 (16.4-19.6)  0 | 13.7 (12.7-14.8)  0 |
| **Health status measures** |  |  |
| KOOS-12 pain subscale, mean (95% CI)  Missing, n | 46.1 (45.5-46.7)  0 | 52.8 (52.2-53.3)  0 |
| KOOS-12 function subscale, mean (95% CI)  Missing, n | 51.6 (50.9-52.3)  0 | 60.0 (59.4-60.5)  0 |
| KOOS-12 quality of life subscale, mean (95% CI)  Missing, n | 42.0 (41.4-42.6)  0 | 48.3 (47.8-48.8)  0 |
| ASES pain subscale, mean (95% CI)  Missing, n | 60.6 (59.8-61.4)  0 | 67.2 (66.6-67.9)  10 |
| ASES other symptoms subscale, mean (95% CI)  Missing, n | 64.5 (63.8-65.3)  1 | 71.8 (71.2-72.4)  9 |
| UCLA Activity Score, mean (95% CI)  Missing, n | 5.4 (5.3-5.5)  0 | 5.5 (5.5-5.6)  0 |
| 30-second chair-stand test, mean (95% CI)  Missing, n | 11.6 (11.4-11.8)  116 | 12.1 (12.0-12.2)  223 |
| 40-meter fast-paced walk test, mean (95%)  Missing, n | 29.8 (29.4-30.2)  153 | 28.5 (28.2-28.8)  257 |

*KOOS-12 (all subscales) scored 0(worst) to 100(best); ASES (all subscales) scored 10(worst) to 100(best); UCLA Activity Score scored 1(inactive) to 10(active); 30-second chair-stand test scored as number of repetitions completed; 40-meter fast-paced walk test scored in seconds.

**Table S2.** Baseline characteristics for hip osteoarthritis participants with and without comorbid lumbar spinal stenosis symptoms based on primary analysis outcome definition.

|  | **Symptoms of LSS** (n=1,253) | **No symptoms of LSS** (n=1,342) |
| --- | --- | --- |
| **Social demographics** |  |  |
| Age, % (95% CI)  <50  50-59  60-69  70-79  ≥80  Missing, n | 5.1 (4.0-6.5)  20.6 (18.4-22.9)  36.2 (33.6-39.0)  32.9 (30.3-35.6)  5.2 (4.0-6.6)  0 | 3.6 (2.6-4.7)  14.1 (12.3-16.1)  35.2 (32.7-37.9)  39.9 (37.3-42.6)  7.2 (5.8-8.7)  0 |
| Female, % (95% CI)  Missing, n | 68.3 (65.7-70.9)  0 | 69.4 (66.9-71.9)  0 |
| Body mass index, % (95% CI)  Underweight  Healthy weight  Overweight  Obese  Missing, n | 0.5 (0.2-1.0)  33.4 (30.8-36.0)  38.1 (35.4-40.8)  27.0 (24.5-29.5)  14 | 1.0 (0.5-1.7)  37.0 (34.4-39.7)  38.7 (36.1-41.4)  22.7 (20.4-25.0)  8 |
| Education level, % (95% CI)  Primary school  Secondary school  Short-term education  Middle-term education  Long-term education  Missing, n | 18.0 (15.9-20.3)  11.3 (9.6-13.2)  21.5 (19.2-23.8)  38.5 (35.8-41.2)  10.5 (8.9-12.4)  2 | 17.5 (15.5-19.7)  11.9 (10.2-13.8)  19.4 (17.3-21.6)  38.7 (36.1-41.4)  12.2 (10.5-14.1)  3 |
| Current employment, % (95% CI)  Employed/student  Sick leave full-time  Sick leave part-time  Retired  Unemployed  Self-imposed early retirement  Early retirement due to low workability  Missing, n | 32.4 (29.8-35.1)  1.7 (1.0-2.6)  3.2 (2.4-4.4)  54.7 (51.9-57.5)  1.9 (1.2-2.8)  3.4 (2.5-4.6)  2.6 (1.8-3.6)  0 | 27.1 (24.8-29.6)  1.4 (0.9-2.2)  1.3 (0.7-2.0)  63.6 (61.0-66.2)  0.8 (0.4-1.5)  3.4 (2.5-4.5)  2.3 (1.6-3.3)  0 |
| Sick leave in past year, % (95% CI)  Missing, n | 9.3 (7.7-11.0)  0 | 4.2 (3.2-5.4)  0 |
| **Clinical characteristics** |  |  |
| Symptom duration, % (95%CI)  <3 months  3-12 months  13-24 months  >24 months  Missing, n | 3.7 (3.7-4.9)  46.4 (43.7-49.3)  20.4 (18.2-22.7)  29.5 (27.0-32.1)  0 | 7.1 (5.8-8.6)  48.7 (46.0-51.4)  19.1 (17.1-21.4)  25.1 (22.8-27.5)  0 |
| Bilateral hip symptoms, % (95% CI)  Missing, n | 28.3 (25.8-30.8)  0 | 21.2 (19.0-23.4)  0 |
| Comorbid knee symptoms, % (95% CI)  Missing, n | 38.1 (35.4-40.9)  0 | 32.3 (29.7-34.8)  0 |
| Back pain in last month, % (95% CI)  Missing, n | 81.6 (79.4-83.6)  0 | 68.9 (66.3-71.3)  0 |
| Number of comorbidities, % (95% CI)  None  One  Two  Three or more  Missing, n | 32.3 (29.7-35.0)  34.4 (31.8-37.1)  20.9 (18.7-23.3)  12.2 (10.4-14.2)  2 | 37.3 (34.7-40.0)  37.7 (35.1-40.4)  17.6 (15.6-19.7)  7.4 (6.0-8.9)  0 |
| Pain medication use, % (95% CI)  Missing, n | 70.9 (68.3-73.4)  0 | 60.6 (57.9-63.2)  0 |
| Opioid use, % (95% CI)  Missing, n | 8.7 (7.3-10.5)  0 | 4.8 (3.7-6.0)  0 |
| Fear of movement, % (95% CI)  Missing, n | 12.3 (10.5-14.2)  0 | 8.3 (6.9-10.0)  0 |
| **Health status measures** |  |  |
| HOOS-12 pain subscale, mean (95% CI)  Missing, n | 46.2 (45.4-47.1)  0 | 52.2 (51.3-53.1)  0 |
| HOOS-12 function subscale, mean (95% CI)  Missing, n | 55.4 (54.3-56.5)  0 | 63.1 (62.1-64.2)  0 |
| HOOS-12 quality of life subscale, mean (95% CI)  Missing, n | 45.7 (44.8-46.6)  0 | 51.7 (50.8-52.6)  0 |
| ASES pain subscale, mean (95% CI)  Missing, n | 57.8 (56.6-58.9)  2 | 64.5 (63.3-65.6)  3 |
| ASES other symptoms subscale, mean (95% CI)  Missing, n | 63.5 (62.4-64.5)  2 | 70.3 (69.3-71.3)  3 |
| UCLA Activity Score, mean (95% CI)  Missing, n | 5.5 (5.4-5.7)  0 | 5.6 (5.5-5.7)  0 |
| 30-second chair-stand test, mean (95% CI)  Missing, n | 11.9 (11.7-12.2)  61 | 12.4 (12.2-12.6)  62 |
| 40-meter fast-paced walk test, mean (95%)  Missing, n | 29.0 (28.5-29.5)  85 | 28.5 (28.1-28.9)  82 |

*HOOS-12 (all subscales) scored 0(worst) to 100(best); ASES (all subscales) scored 10(worst) to 100(best); UCLA Activity Score scored 1(inactive) to 10(active); 30-second chair-stand test scored as number of repetitions completed; 40-meter fast-paced walk test scored in seconds.
